# Safety and Efficacy of Maxitrol in Pediatric Age Group Below Two Years With Adenoid Hypertrophy: A Retrospective Cohort Study

**DOI:** 10.7759/cureus.14736

**Published:** 2021-04-28

**Authors:** Mohammed Halawani, Abdullah Alkhaldi, Abdullah Almajed, Ahmed Almutairi, Maali Alrashed, Nouf Albakeet, Wala Alshiha, Omar Aldibasi, Jaber Alshammari

**Affiliations:** 1 Otolaryngology Head & Neck Surgery, King Abdullah Specialized Children Hospital, King Abdulaziz Medical City, National Guard Health Affairs, Riyadh, SAU; 2 Medicine, King Saud Bin Abdulaziz University for Health Sciences, Riyadh, SAU; 3 Biostatistics and Bioinformatics, King Abdullah International Medical Research Center (KAIMRC), Riyadh, SAU

**Keywords:** adenoid hypertrophy, intranasal corticosteroids, maxitrol, intranasal steroids safety

## Abstract

Introduction

Adenoid hypertrophy, a common condition in children, represents one of the common indications for surgery in pediatrics. Medical treatment alone is not effective, and most of the time patients are managed by surgical removal of the adenoid. The aim of this study is to assess the safety and efficacy of intranasal Maxitrol® drops (Novartis Pharmaceuticals, Basel, Switzerland) in pediatric patients with adenoid hypertrophy aged less than two years and to document any side effects during its use.

Methods

This retrospective cohort study was conducted at King Abdullah Specialist Children’s Hospital (KASCH). We reviewed the charts of 86 pediatric patients aged less than two years who were diagnosed with adenoid hypertrophy between 2015 and 2018. Patients were grouped according to the type of intervention (use of Maxitrol®, and no use). The follow-up time was up to one year.

Results

Out of 86 patients, 55 (63.9%) patients had adenoid hypertrophy alone and 31 (36.1%) had adenoid hypertrophy plus another disease. Patients with obstructive sleep apnea symptoms (p=0.026) and grade of adenoid (p=0.040) showed a significant relationship with surgery booking after one year. The probability of booking for surgery for those who used Maxitrol® was 1.394 times higher than for those who were not using it (odds ratio [OR]=1.394; 95% confidence interval [CI]=0.549-3.537). Suppression of growth and eye complications were not reported in any of our patients.

Conclusion

In this small sample, the use of Maxitrol® in the pediatric age group below two years with adenoid hypertrophy was safe and effective in relieving nasal symptoms; however, eventually, surgery was needed in most of our patients. Suppression of growth and eye complications were not reported in any of our patients during the follow-up time.

## Introduction

Adenoids are pyramid-shaped lymphoid tissue located in the nasopharynx [1]. Adenoid hypertrophy is a common disease in pediatrics. It usually presents with symptoms of airway obstruction such as mouth breathing and snoring [2]. Adenoid hypertrophy treatment in pediatrics is determined by the degree of airway obstruction and related comorbidities. Most of the time, it is treated by adenoidectomy. However, medical therapy is crucial and must be tried before surgical intervention. Various studies have been published about the use of intranasal corticosteroids in children, and they have shown promising results [3-8].

Intranasal corticosteroids have been used in pediatric patients aged less than two years at King Abdullah Specialist Children’s Hospital to decrease nasal obstruction symptoms secondary to rhinitis and adenoid hypertrophy. They were the first line of therapy in patients with allergic rhinitis in the past few decades. However, they are not recommended for use in pediatric patients younger than two years due to a lack of evidence on their safety in this age group.

To our knowledge, the use of intranasal Maxitrol® in pediatric patients below two years with adenoidal hypertrophy has not been investigated in the literature. Our study aims to assess intranasal Maxitrol’s safety and efficacy in pediatric patients with adenoid hypertrophy aged less than two years and document any side effects during its use, such as eye complications and suppression of growth.

## Materials and methods

This retrospective cohort study was conducted at King Abdullah Specialist Children’s Hospital, Riyadh, Saudi Arabia. We reviewed the charts and clinical information of pediatric patients aged less than two years who were diagnosed with adenoid hypertrophy between 2015 and 2018. The inclusion criteria were patients aged less than two years with adenoid hypertrophy as the primary disease and complete follow-up. We excluded patients who were using steroids (systemic/nebulizer) and patients with missing data. We reviewed 123 pediatric patients’ files from our electronic healthcare system (BESTcare); however, only 86 patients met our inclusion criteria. Out of 86 patients, 47 used Maxitrol®, while 39 did not take Maxitrol (control group). In our study, Maxitrol® (a sterile drop containing the active ingredients neomycin, polymyxin b, and dexamethasone) was used as two drops in each nostril per day for two to six weeks depending on symptoms improvement. The follow-up time was up to one year. The institutional review board approved this study at King Abdullah International Medical Research Center (RC20/016/R).

Statistical analyses were performed using SAS 9.4 (SAS Institute Inc., Cary, NC, USA), and the level of significance was declared at α=.05. Categorical variables were presented as percentages, and frequencies continuous variables were reported in terms of means and standard deviations. Patient-specific characteristics included age, gender, height and weight, and the use of Maxitrol®. Patients were grouped according to the type of intervention (use of Maxitrol®, and no use) and compared across other variables using univariate analysis, including χ2 tests and Student t-test. Outcomes obtained included the date of surgery, local eye symptoms, effect on growth parameters, systemic symptoms, delay of surgery, and mortality.

Using booking for surgery within one year as an outcome variable, we compared the group who were booked for surgery within one year to the group who were not booked across other variables using univariate analysis, including χ2 tests and Student t-test. Further, a logistic regression model was applied to model the probability of booking surgery within one year.

## Results

We analyzed 89 patients to examine the efficacy and safety of Maxitrol® in children below two years old with adenoid hypertrophy. As seen in Table 1, males were dominant (87.2%), with nearly two-thirds (63.9%) diagnosed with adenoid hypertrophy alone. Furthermore, 19.8% were presented without obstructive sleep apnea symptoms. The proportions of patients who reported snoring, sleep disturbance, and apnea were 15.1%, 43%, and 66.7%, respectively. The most commonly diagnosed grade of tonsil was grade 1 (45.3%), followed by grade 2 (40.7%). Likewise, 10.5% presented with asthma and 3.5% with Down syndrome. Furthermore, none of the patients presented with any eye complications. The mean height (cm), weight (kg), and BMI (kg/m2) of the patients were 77.7, 9.89, and 16.5, respectively. Comparing the baseline characteristics of the patients in relation to the use of Maxitrol® revealed that male gender (p<0.001), adenoid hypertrophy plus other diseases (p=0.007), and those without syndrome (p=0.028) were significantly more associated with the use of Maxitrol®.

**Table 1:** Demographics and baseline clinical characteristics of all patients in relation to the use of Maxitrol® BMI: body mass index; OSA: obstructive sleep apnea

Variables	Levels	Used Maxitrol® (n=47) N(%)	No Maxitrol® (n=39) N(%)	total	P-value
Height at the first visit		77.74 +8.9	77.71 +7.39	77.72 +8.2	0.9870
Weight at the first visit		9.99 +2.36	9.77 +1.68	9.89 +2.07	0.6307
BMI at first visit		16.51 +2.57	16.49 +2.11	16.5 +2.36	0.9666
Gender	Male	47 (62.67)	28 (37.33)	75(87.20)	< .0001>
	Female	0 (0.00)	11 (100.00)	11 (12.79)	
Diagnosis	Adenoid hypertrophy	24 (43.64)	31 (56.36)	55 (63.95)	0.0073
	Adenoid hypertrophy+ other disease	23 (74.19)	8 (25.81)	31 (36.05)	
OSA symptoms	No OSA symptoms	5 (29.41)	12 (70.59)	17 (19.77)	0.1265
	Snoring	8 (61.54)	5 (38.46)	13 (15.12)	
	Sleep disturbance/mouth breathing	21 (56.76)	16 (43.24)	37 (43.02)	
	Apnea/Cyanosis	12 (66.67)	6 (33.33)	18 (20.93)	
Grade of tonsil	Grade 1 tonsil	17 (43.59)	22 (56.41)	39 (45.35)	0.1992
	Grade 2 tonsil	22 (62.86)	13 (37.14)	35 (40.7)	
	Grade 3 tonsil	6 (60.00)	4 (40.00)	10 (11.63)	
	Grade 4 tonsil	2 (100.00)	0 (0.00)	2 (2.33)	
Grade of adenoid	Grade 1 adenoid	7 (50.00)	7 (50.00)	14 (16.28)	0.4304
	Grade 2 adenoid	13 (46.43)	15 (53.57)	28 (32.56)	
	Grade 3 adenoid	27 (61.36)	17 (38.64)	44 (51.16)	
Asthma	No asthma	42 (54.55)	35 (45.45)	77 (89.53)	1.0000
	Asthma	5 (55.56)	4 (44.44)	9 (10.47)	
Syndromes	No syndrome	45 (60.00)	30 (40.00)	75 (87.21)	0.0280
	Down syndrome	1 (33.33)	2 (66.67)	3 (3.49)	
	Other syndromes	1 (12.50)	2 (66.67)	8 (9.3)	
Eye complications	No eye complications	47 (54.65)	39 (45.35)	86 (100)	

Table 2 shows the relationship between the baseline characteristics and surgery booking after one year. The results reveal that obstructive sleep apnea (OSA) symptoms (p=0.026) and adenoid grade (p=0.040) show a significant relationship with surgery booking after one year. Other baseline characteristics, including height, weight, BMI, gender, diagnosis, tonsil grade, asthma, syndrome, and eye complications, did not show a significant relationship compared to surgery booking after one year (all p>0.05).

**Table 2:** Demographics and baseline clinical characteristics of all patients in relation to surgery booking after one year BMI: body mass index; OSA: obstructive sleep apnea

Variables	Levels	No surgery within 1 year (n=45) N(%)	Booked for surgery (n=41) N(%)	Total	P-value
Height at the first visit		76.84 +8.49	78.72 +7.84	77.72 +8.2	0.2921
Weight at the first visit		9.78 +2.15	10.02 +2.01	9.89 +2.07	0.5843
BMI at first visit		16.83 +2.54	16.13 +2.12	16.5 +2.36	0.1751
Gender	Male	38 (50.67)	37 (49.33)	75(87.20)	0.5259
	Female	7 (63.64)	4 (36.36)	11 (12.79)	
Diagnosis	Adenoid hypertrophy	30 (54.55)	25 (45.45)	55 (63.95)	0.6557
	Adenoid hypertrophy+ other disease	15 (48.39)	16 (51.61)	31 (36.05)	
OSA symptoms	No OSA symptoms	12 (70.59)	5 (29.41)	17 (19.77)	0.0258
	Snoring	8 (61.54)	5 (38.46)	13 (15.12)	
	Sleep disturbance/mouth breathing	20 (54.05)	17 (45.95)	37 (43.02)	
	Apnea/Cyanosis	4 (22.22)	14 (77.78)	18 (20.93)	
Grade of tonsil	Grade 1 tonsil	19 (48.72)	20 (51.28)	39 (45.35)	0.3978
	Grade 2 tonsil	20 (57.14)	15 (42.86)	35 (40.7)	
	Grade 3 tonsil	6 (60.00)	4 (40.00)	10 (11.63)	
	Grade 4 tonsil	0 (0.00)	2 (100.00)	2 (2.33)	
Grade of adenoid	Grade 1 adenoid	11 (78.57)	3 (21.43)	14 (16.28)	0.0403
	Grade 2 adenoid	16 (57.14)	12 (42.86)	28 (32.56)	
	Grade 3 adenoid	18 (40.91)	26 (59.09)	44 (51.16)	
Asthma	No asthma	42 (54.55)	35 (45.45)	77 (89.53)	0.2993
	Asthma	3 (33.33)	6 (66.67)	9 (10.47)	
Syndromes	No syndrome	38 (50.67)	37 (49.33)	75 (87.21)	0.7182
	Down syndrome	2 (66.67)	1 (33.33)	3 (3.49)	
	Other syndromes	5 (62.50)	3 (37.50)	8 (9.3)	
Eye complications	No eye complications	45 (52.33)	41 (47.67)	86 (100)	

The relationship between the use of Maxitrol® and booking for surgery was not statistically significant (p=0.26) as Table 3 shows. There was no significant difference in terms of surgery booking with Maxitrol® use. Patients without OSA symptoms were less likely to book a surgery than were those with apnea/cyanosis (odds ratio [OR]=0.133; 95% confidence interval [CI]=0.025-0.630). Patients with sleep disturbance/mouth breathing are less likely to book surgery than are those with apnea/cyanosis (OR=0.249; 95% CI=0.069-0.873). Moreover, patients who reported snoring are less likely to book surgery than are those with apnea/cyanosis (OR=0.037-0.873) (Table 4).

**Table 3:** Outcome of the use of Maxitrol®

Use of Maxitrol®	No surgery within 1 year	Booked for surgery	Total	P-value
No Maxitrol	23 (58.97)	16 (41.03)	39	0.2608
Used Maxitrol	22 (46.81)	25 (53.19)	47	
Total	45	41	86	

**Table 4:** Associated factors with surgery booking in terms of OR based on logistic regression model (modeling the probability of surgery booking) OSA: obstructive sleep apnea; OR: odds ratio; CI: confidence interval

Effect	Levels	OR	95% CI
Use of Maxitrol®	No	Ref.	
	Yes	1.394	(0.549, 3.537)
OSA symptoms	Apnea / Cyanosis	Ref.	
	No Symptom	0.133	(0.028, 0.63)
	Sleep disturbance/mouth breathing	0.249	(0.069, 0.904)
	Snoring	0.18	(0.037, 0.873)

## Discussion

Intranasal corticosteroids treatment in children with adenoid hypertrophy were first introduced by Demain in 1995 [3]. The mechanism through which intranasal corticosteroids relieve nasal airway obstruction symptoms has not yet been determined. However, it is thought that they decrease adenoid hypertrophy through their lympholytic or anti-inflammatory effects [6].

A study about the spread of topical corticosteroid sprays in the nasal cavity showed that the spread is insufficient [9,10]. On the other hand, topical intranasal steroid in drop form showed more spread in the nasal cavity and reached the nasopharynx faster.

It was demonstrated that the medical use of intranasal corticosteroids such as fluticasone propionate [11] or mometasone furoate [12] for the treatment of adenoid hypertrophy showed significant results in older children. Zhang et al., in their meta-analysis, showed a five-fold reduction in adenoid size after treatment with intranasal corticosteroids [13]. In another meta-analysis, Chohan et al. evaluated intranasal mometasone furoate in terms of nasal symptoms, adenoid size, or adenoid/choana ratio, and stated that it was effective [14]. Additionally, three months of treatment for adenoid hypertrophy with intranasal corticosteroids and antihistamines significantly reduced adenoid size [15].

There is no consensus on the duration of treatment and optimal dose of intranasal corticosteroids. In our study, Maxitrol® was prescribed as two drops in each nostril daily for two to six weeks depending on symptoms improvement. Maxitrol® succeeded to improve nasal symptoms (i.e., snoring and nasal discharge); however, it failed to treat adenoid hypertrophy. As a result, surgery was eventually needed in most of our patients. Furthermore, the probability of booking for surgery for our patients who used Maxitrol® was 1.394 times higher than for those who were not using it. While Varricchio et al. demonstrated lower rates of adenoidectomy following intranasal flunisolide treatment [16], we found that the relationship between the use of Maxitrol® and booking for surgery was not statistically significant in our study. We observed that the probability of booking for surgery for those with sleep disturbance and mouth breathing (OSA symptoms) was likely to increase.

Topical corticosteroids have fewer side effects than do oral corticosteroids. The local side effects are mainly dry nose, bleeding, crusting, and candidiasis [17]. They are less likely to cause systemic effects on bone metabolism, effects on growth, and the adrenocortical axis [18]. Skoner et al. demonstrated the negative effect of intranasal beclomethasone dipropionate on growth after one year in pediatric patients (aged six to nine years) as it showed significant suppression of growth [19]. On the contrary, other studies have shown no suppression of bone growth in children after one year of treatment with a pediatric dose of mometasone furcate (MF) nasal spray (100 µg daily) or with budesonide (BUD) (200 µg twice daily) [20,21]. Similarly, the use of Maxitrol® in our study had no effect on growth parameters in our patients when they were seen at their follow-up time after one year.

The age restriction for the use of intranasal corticosteroids is variable; for fluticasone furoate and mometasone, it is two years and older [22]. Mometasone furoate was found to be the most reliable drug in children older than two years [23,24]. None of the other studies have investigated, as we did, the use of intranasal corticosteroids in children younger than two years. Alhussien et al. suggested that intranasal use of fluticasone furoate, mometasone, and budesonide is safe if they are used at the recommended therapeutic dose after a proper medical evaluation during pregnancy [25]. Such a study can raise the question of whether intranasal steroids are safe during fetal periods and why they might be contraindicated during the first two years of life.

## Conclusions

In this small sample, the use of Maxitrol® in the pediatric age group below two years with adenoid hypertrophy was safe and effective in relieving nasal symptoms; however, surgery was needed in most of our patients. Eye complications and suppression of growth were not reported in any of our patients during the follow-up time after one year. Further long-term large randomized clinical trials are needed to evaluate the safety and efficacy of Maxitrol®.
